# Triglyceride-Rich Lipoproteins and Glycoprotein A and B Assessed by 1H-NMR in Metabolic-Associated Fatty Liver Disease

**DOI:** 10.3389/fendo.2021.775677

**Published:** 2022-01-10

**Authors:** Juan Moreno-Vedia, Roser Rosales, Enrique Ozcariz, Dídac Llop, Maribel Lahuerta, María Benavent, Ricardo Rodríguez-Calvo, Núria Plana, Angels Pedragosa, Lluís Masana, Antoni Castro, Daiana Ibarretxe, Josefa Girona

**Affiliations:** ^1^ Vascular Medicine and Metabolism Unit, Research Unit on Lipids and Atherosclerosis, Sant Joan University Hospital, Universitat Rovira i Virgili, Reus, Spain; ^2^ Institut Investigació Sanitaria Pere Virgili (IISPV), Reus, Spain; ^3^ Spanish Biomedical Research Centre in Diabetes and Associated Metabolic Disorders (CIBERDEM), Madrid, Spain; ^4^ Biosfer Teslab SL, Reus, Spain; ^5^ Internal Medicine Department, Sant Joan University Hospital, Universitat Rovira i Virgili, Reus, Spain

**Keywords:** triglyceride-rich lipoproteins, glycoproteins, NMR, metabolic-associated fatty liver disease, cardiovascular risk

## Abstract

High plasma triglyceride (TG) levels and chronic inflammation are important factors related to metabolic-associated fatty liver disease in patients at cardiovascular risk. Using nuclear magnetic resonance (^1^H-NMR), we aimed to study the triglyceride-rich lipoprotein (TRL) and acute-phase glycoprotein profiles of a cohort of patients with metabolic disease and their relationship with fatty liver. Plasma samples of 280 patients (type 2 diabetes, 81.1%; obesity, 63.3%; and metabolic syndrome, 91.8%) from the University Hospital Lipid Unit were collected for the measurement of small, medium and large TRL particle numbers and sizes and glycoprotein profiles (Glyc-A and Glyc-B) by ^1^H-NMR. Liver function parameters, including the fatty liver index (FLI) and fibrosis-4 (FIB-4) score, were assessed. Hepatic echography assessment was performed in 100 patients, and they were followed up for 10 years. TRL particle concentrations showed a strong positive association with Glyc-A and Glyc-B (ρ=0.895 and ρ=0.654, p<0.001, respectively) and with the liver function-related proteins ALT ρ=0.293, p<0.001), AST (ρ=0.318, p<0.001) and GGT (ρ=0.284, p<0.001). Likewise, TRL concentrations showed a positive association with FLI (ρ=0.425, p<0.001) but not with FIB-4. During the follow-up period of 10 years, 18 new cases of steatosis were observed among 64 patients who were disease-free at baseline. Baseline TRL particle numbers and glycoprotein levels were associated with the new development of metabolic-associated fatty liver disease (MAFLD) (AUC=0.692, p=0.018 and AUC=0.669, p=0.037, respectively). Overall, our results indicated that TRL number and acute-phase glycoproteins measured by ^1^H-NMR could be potential biomarkers of the development of hepatic steatosis in patients at metabolic risk.

## Introduction

Triglycerides (TGs) are important cardiovascular risk factors (1). These lipids are transported mainly by triglyceride-rich lipoproteins (TRLs), a group of circulating lipoproteins that include chylomicrons (only present postprandially or in pathological conditions), VLDL and their remnants, and IDL. Therefore, TRLs are a group of lipoproteins with a high TG content. Increases in TG levels and, thus, in TRL particle numbers, are associated with cardiovascular risk and metabolic disorders (2, 3).

Hypertriglyceridemia is a common component of metabolic alterations such as type 2 diabetes (T2DM), metabolic syndrome and obesity. Under these conditions, the development of insulin resistance leads to hepatic TG accumulation due to an enhanced FA uptake into the liver and an increased *de novo* lipogenesis, enhancing TG availability. The imbalance between VLDL oversynthesis and the secretion of larger VLDL_1_ particles in the abovementioned metabolic diseases leads to both ectopic hepatic fat accumulation and higher plasma TG concentrations, leading to fatty liver disease (3, 4). These metabolic alterations are also associated with a low degree of chronic systemic inflammation along with local liver inflammation secondary in part to lipotoxicity. These highly prevalent conditions lead to metabolic-associated fatty liver diseases (MAFLDs), cirrhosis or even liver cancer, among others (5, 6).

Recently, as previously discussed (7), MAFLD has become a major cause of chronic liver disease worldwide, and it has become a challenge to public health, with an estimated prevalence of 25% worldwide and 23% in Europe (8). It is defined as the accumulation of fat in the liver in the presence of metabolic dysfunction and can range from simple steatosis, with or without mild nonspecific inflammation, to steatohepatitis characterized by the presence of inflammation and hepatocyte damage that can eventually lead to progressive fibrosis and cirrhosis (9, 10). Considering the vital role of the liver in lipid metabolism (including the uptake and secretion of plasma lipoproteins) (11) and its central role in the inflammatory cascade, hepatic alterations could be expected as a consequence of a liver overloaded with fat. Moreover, all components of metabolic syndrome (Met-S) correlate with liver fat content, with insulin resistance being one of the main pathogenic factors of hepatic fat accumulation. Such metabolic comorbidities are known to generate multiple signals in an inflammatory environment/status that can contribute to liver damage (12, 13).

New biomarkers, such as plasma acute-phase glycoproteins, which can be detected by ^1^H-NMR as glycoproteins A and B, have emerged as promising tools to detect inflammatory patterns. Human plasma acute-phase glycoproteins are synthesized by the liver and induced by inflammatory cytokines such as IL-1, IL-6 or tumor necrosis factor, representing a systemic inflammatory response (14). They are almost all N-linked glycoproteins containing oligosaccharide chains attached to asparagine residues (15). ^1^H-NMR detects the signal produced by the acetyl groups (-COCH3) of N-acetylglucosamine and N-acetylgalactosamine (Glyc-A) and N-acetylneuraminic acid (Glyc-B). Glyc-A and B are composite biomarkers that integrate the protein levels and glycation states of several of the most abundant acute phase proteins in the serum, including alpha-1-acid glycoprotein (AGP), alpha-1-antitrypsin (AAT), alpha-1-antichymotrypsin (AACT), haptoglobin, and transferrin. This makes them a more stable measure of inflammatory status with less intraindividual variability than other biomarkers, such as hsCRP (16, 17).

Glycoproteins A and B have been reported to be elevated in subclinical chronic inflammatory states such as obesity, diabetes, chronic infections or autoimmune diseases and are known to be strong biomarkers of cardiovascular diseases (18–20). The detection of glycation patterns, as well as the determination of acute-phase glycoprotein serum concentrations, has provided new insights into the field of liver disease, with special interest in their possible applications to distinguish the presence of inflammation during the course of the disease (21–24). In this field, ^1^H-NRM has emerged as a promising strategy to measure plasma levels of glycoprotein-related signals and patterns (16).

In the present study, we aimed to describe the type of TRL particle fraction and the acute phase protein profile, both determined by ^1^H-NMR in a cohort of dysmetabolic patients and their association with hepatic damage. Considering the inflammatory status related to metabolic syndrome and hepatic steatosis, we investigated the possible role of glycoproteins A and B in the detection of hepatic disease and their possible predictive capacities over a 10-year follow-up.

## Materials and Methods

### Design and Study Subjects

We performed a baseline cross-sectional and a retrospective/prospective study at the 10-year follow-up.

At baseline, we included 280 patients who were willing to participate that were attending the Lipid Unit of our University Hospital due to lipid metabolism disturbances and associated disorders such as obesity, T2DM and Met-S. Obesity, T2DM and Met-S were diagnosed according to standard clinical criteria. Subjects with chronic lung or renal diseases or cancer were excluded. Patients on lipid-lowering drugs underwent a 6-week wash-out period (8 weeks if they were on fibrates). Anamnesis, anthropometric, and physical examination data were recorded. Liver ultrasound (i.e., greyscale abdominal ultrasound evaluation of the liver) was performed at baseline for 100 patients to evaluate the presence of hepatic steatosis. Hepatic steatosis was defined by an increased echogenicity of the hepatic parenchyma, which provides a brighter image than the kidney’s cortex (25).

For the prospective study, we studied the association between the baseline data and liver ultrasound echography data obtained after 10 years of follow-up for 64 patients who were free of hepatic steatosis at baseline.

This study was approved by the Ethical and Clinical Investigation Committee of the Pere Virgili Institute for Health Research (IISPV) and fulfilled the principles of the Helsinki Declaration. A written consent form was signed by all participants.

### Non-Invasive Fatty Liver Disease Indexes

The fatty liver index (FLI) was calculated based on the body mass index (BMI), waist circumference, triglycerides and γ-glutamyltransferase (GGT) using the following formula:


$$FLI=\frac{(e^{0.953\times \log\;e(triglycerides)+0.139\times BMI+0.718\times \log\;e(GGT)+0.053\times waistcircumferecence-15.745)}}{1+(e^{0.953\times \log\;e(triglycerides)+0.139\times BMI+0.718\times \log\;e(GGT)+0.053\times waistcircumferecence-15.745)}}\times 100$$

as described previously (26). The fibrosis 4 score (FIB4) was calculated based on age (years), AST and ALT levels (U/L), and platelet counts (10^9^/L) using the following formula (27):


$$FIB4=\frac{Age\times AST}{Platelet\;count\times \sqrt{ALT}}$$

### Clinical and Standard Biochemical Analysis

Anamnesis and anthropometric data, including sex, age, clinical history and medication, were recorded and included in our database. BMI was calculated from the weight and height measurements (kg/m^2^). A blood sample was obtained from each patient after overnight fasting. Aliquots were prepared for immediate storage at -80°C in the BioBank at our centre prior to use. Standard biochemical parameters, including lipids, apolipoproteins, blood glucose, hsCRP, and transaminases, were measured using colorimetric, enzymatic and immunoturbidimetric assays (Spinreact, SA, Spain; Horiba, SA, Spain), which were adapted to the Cobas Mira Plus Autoanalyser (Roche Diagnostics, Spain).

### TRL Particle Analysis by ^1^H-NMR

The TRL particle number and size were assessed by the Liposcale test^®^, which is a new generation 2D-^1^H-NMR test developed with the collaboration of our group (28). In brief, 200 µl of serum was diluted with 50 µl of deuterated water and 300 µl of 50 mM phosphate buffer solution (PBS) at pH 7.4. ^1^H-NMR spectra were recorded at 305.95 K on a Bruker Avance III 600 spectrometer operating at a proton frequency of 600.20 MHz (14.1 T). The particle size (Z) and particle number concentration (P) of three subtypes of TRLs (including large, medium, small and total TRLs) were analysed. Particle concentrations and diffusion coefficients were obtained from the measured distinct methyl groups of the 2D ^1^H-NMR spectra after the deconvolution analysis of the signals of the NMR pulse. The methyl signal was surface fitted with the Lorentzian functions associated with each lipoprotein subtype. The area of each Lorentzian function reflected the lipid concentration of each subtype, and the size of each subtype was calculated from the diffusion coefficient. The particle number of each TRL subtype was calculated by dividing the lipid volume by the particle volume of a given class. Lipid volumes were determined using common conversion factors to convert the concentration units into volume units. The variation coefficients for particle number were between 2% and 4%. The variation coefficients for particle size were lower than 0.3%.

### Glycoprotein Analysis by ^1^H-NMR

The same processing prior to NMR analysis was performed for plasma glycoprotein analysis, following previously reported procedures (18). Briefly, the region of the ^1^H-NMR spectrum where the glycoproteins resonate (2.15–1.90 ppm) was analysed using several functions according to the chemical shift: Glyc-A and Glyc-B. For each function, we determined the total area and transformed it to concentration according to the number of sugar–protein bonds. The area, height, position, and bandwidth and their ratios were also calculated. The concentrations of Glyc-A and Glyc-B provided the amount of acetyl groups of protein bond N-acetylglucosamine, N-acetylgalactosamine (Glyc-A), and N-acetylneuraminic acid (Glyc-B), the predominant sialic acid found (16).

### Statistical Analysis

The normality of continuous variables was determined by the Kolmogorov–Smirnov test. Data are presented as the medians and 25^th^ and 75^th^ percentiles (IQR) for continuous variables not normally distributed or the mean and standard deviation (SD) when normally distributed. Categorical variables are expressed as percentages unless otherwise indicated. Differences between groups were evaluated by t-tests or Mann-Whitney U tests. Associations between the variables were analysed by Spearman’s test, partial correlations and univariate regression analysis. Multivariate linear regression analysis was used to analyze the association of TRL-P with glycoproteins. The model included glycoproteins, age, BMI, hsCRP, ALT, AST, GGT, systolic BP, glucose, total cholesterol and sex as covariates. Linear multivariate models were performed in order to study the association between hepatic steatosis with biochemical data, hepatic indexes, TRL lipidomics and inflammation parameters. These associations were adjusted for known confounders to avoid spurious associations. Random forest classification models (RF) were performed based on conditional inference trees to evaluate the importance of each variable in the decision method, which was represented in terms of the mean decrease Gini plot. All statistical analyses were performed using SPSS software (IBM SPSS Statistics, version 27.0.1.0, Madrid, Spain) and R Studio (version 3.6). Statistical tests *p* < 0.05 were defined as significant.

## Results

### Participant’s Characteristics

Our study included 280 patients, with a median age of 61 (52-66) years, of whom 48.9% were female. Type 2 diabetes was present in 81.1%, obesity in 63.3% and Met-S in 91.8% of the participants. **Table 1** summarizes the clinical, anthropometric, and biochemical characteristics of the patients grouped by sex. The women were older than the men (p=0.023). The men had a higher waist circumference, diastolic BP and glucose levels than the women (p<0.05). The plasma lipid profile showed significant differences, with TGs being higher in men (p<0.001) and LDL-C and HDL-C being higher in women (p<0.001). The fatty liver index (FLI, p=0.026) and liver function-related transaminases (p<0.001) were found to be higher in men than in women. All parameters of the TRL lipidomics were higher in men (p<0.05). The women had a higher hsCRP than the men (p=0.017).

**Table 1:** Clinical, anthropometric, and biochemical characteristics of the study population grouped by sex.

	Female n=137	Male n=143	p-value
*Clinical data*			
Age, years	62 (55-67)	58 (50-65)	**0.023**
BMI, kg/m^2^	32.6 (28.7-37.5)	31.1 (29.1-34.8)	0.178
Waist circumference, cm	103 (96-115)	107.5 (102-113)	**0.024**
Systolic BP, mmHg	140 (130-150)	138 (130-152)	0.882
Diastolic BP, mmHg	80 (72-85)	82 (76-89)	**0.029**
Obesity, %	61.3	65.2	0.496
Type 2 diabetes, %	79.6	82.5	0.528
Metabolic syndrome, %	92.0	91.5	0.898
Hypertension, %	61.8	59.3	0.674
*Biochemical data*			
Total cholesterol, mmol/L	5.85 (5.12-6,98)	5.54 (4.75-6.95)	0.177
Triglycerides, mmol/L	1.69 (1.25-2.62)	2.26 (1.48-4.27)	**<0.001**
LDL-C, mmol/L	3.79 ± 1.02	3.31 ± 1.22	**<0.001**
HDL-C, mmol/L	1.26 (1.05-1.38)	0.98 (0.87-1.16)	**<0.001**
Apo B-100, mg/dL	121 (101-145)	115 (97-141)	0.201
Apo A-I, mg/dL	129 (111-149)	115 (101-129)	**<0.001**
Glucose, mg/dL	133 (107-161)	145 (117-178)	**0.016**
HbA_1c_, %	6.3 (5.6-7.45)	6.4 (5.7-7.3)	0.748
AST, U/L	22 (18-28)	26 (21-32)	**<0.001**
ALT, U/L	17 (12-24)	23 (16-35)	**<0.001**
GGT, U/L	23 (16-38)	31 (20-51)	**<0.001**
*Hepatic indexes*			
FLI, %	80.6 (50.9-95.4)	87.3 (72.9-96)	**0.026**
FIB-4	1.66 (1.31-1.96)	1.53 (1.25-1.98)	0.421
*TRL lipidomics*			
Total TRL-P, nmol/L	61.2 (44.3-98.3)	83.2 (52.1-122.9)	**0.004**
Large TRL-P, nmol/L	1.39 (1.04-2.08)	1.86 (1.24-2.78)	**0.001**
Medium TRL-P, nmol/L	7.89 (5.49-12.9)	10.6 (6.34-16.9)	**0.004**
Small TRL-P, nmol/L	52.1 (38.0-82.8)	70.6 (44.1-99.6)	**0.004**
TRL-Z (nm)	42.31 (42.23-42.38)	42.35(42.25-42.44)	**0.007**
*Inflammation parameters*			
Glyc-A, µmol/L	892.1 (769.3-1093.9)	957.4 (795.9-1149.2)	0.149
Glyc-B, µmol/L	367.4 (334.0-401.4)	364 (323.6-419.8)	0.975
hsCRP, mg/L	2.59 (1.57-3.96)	2.05 (1.17-3.44)	**0.017**

Data are the means ± SD for normally distributed variables, medians (IQR) for nonparametric data or n (%). BMI, body mass index; systolic BP, systolic blood pressure; diastolic BP, diastolic blood pressure; LDL-C, LDL cholesterol; HDL-C, HDL cholesterol; Apo B-100, apolipoprotein B100; Apo A-I, apolipoprotein A1; HbA_1c_, glycated hemoglobin; AST, aspartate aminotransferase; ALT, alanine aminotransferase; GGT, gamma-glutamyl transferase; FLI, fatty liver index; FIB-4, fibrosis-4 score; TRL, triglyceride-rich lipoprotein; hsCRP, high-sensitivity C-reactive protein. p values are for group comparisons. Statistical analysis: χ2 for categorical data; t-tests or Mann-Whitney U tests were used for continuous variables. Bold values indicate p < 0.05.

### Association of TRL-P With Plasma Glycoproteins and Hepatic Biomarkers

**Figure 1:**
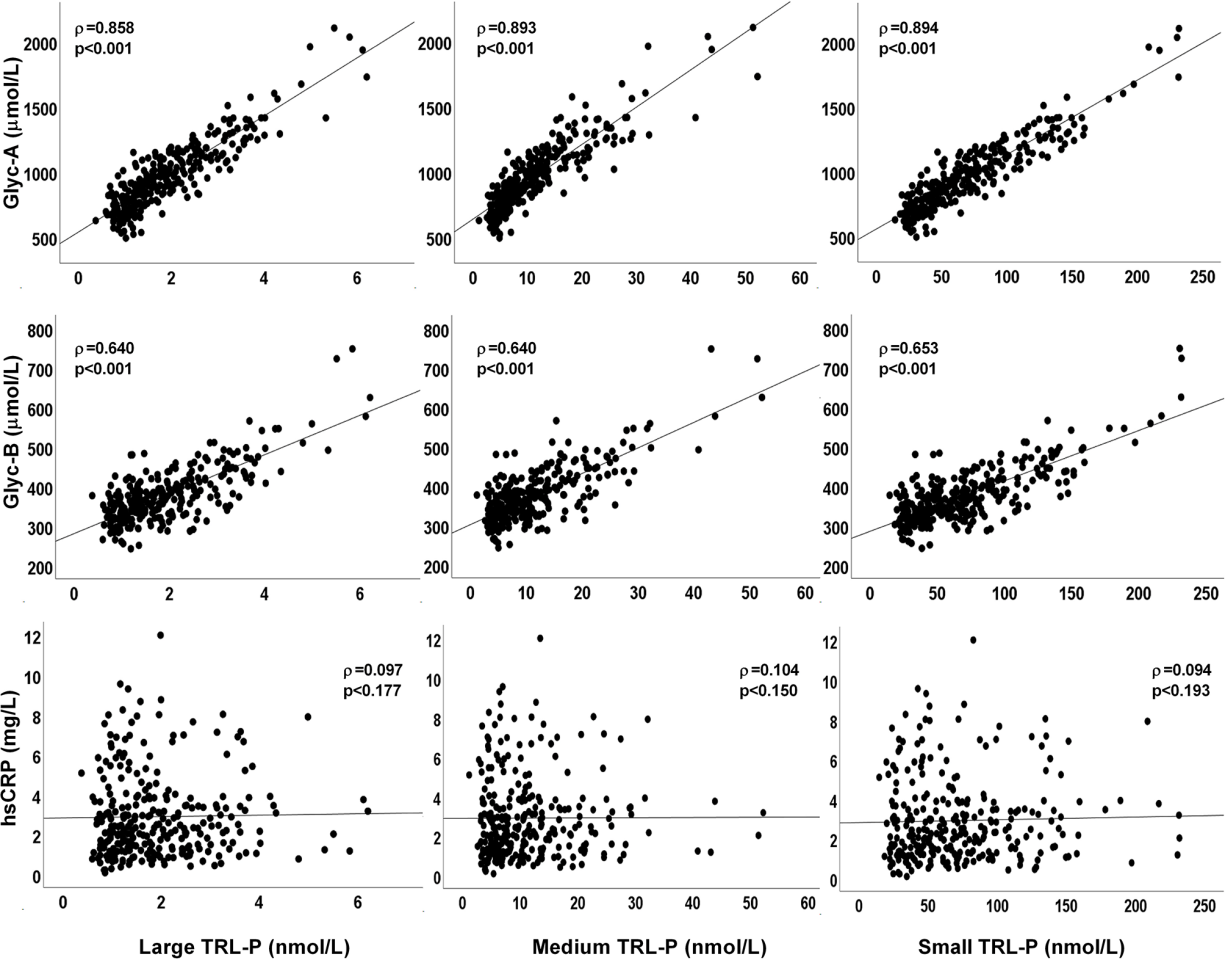
Plots of plasma TRL-P concentrations (large, medium and small particles) with glycoproteins Glyc-A, Glyc-B, and with hsCRP. ρ from Rho coefficients from the Spearman correlation analysis.

Positive correlations were found between total TRL-P and the NMR-measured glycoproteins Glyc-A and Glyc-B (ρ=0.895 and ρ=0.654, p<0.001, respectively). These positive associations were observed between the different TRL subclass particles measured, including large, medium and small particles (**Figure 1**). No correlation was found between total (ρ=0.094, p=0.192) or subclass TRL particles and hsCRP (**Figure 1**).

To further explore the relationship between TRL-P and ^1^H-NMR-measured glycoproteins, a multivariate linear regression analysis that included the variables age, BMI, hsCRP, ALT, AST, GGT, systolic BP, glucose, total cholesterol, sex, Glyc-A and Glyc-B was generated (**Supplementary Table 1**). The significant association between TRL-P and glycoproteins remained robust after adjustment for all covariates (p<0.001).

**Table 2** shows the univariate associations of plasma TG, LDL-C, non-HDL-C, Apo B-100, TRL-P and glycoproteins with the hepatic indexes and profile. TRL-P was significantly positively associated with AST, ALT, GGT and FLI (p<0.05). Additionally, the ^1^H-NMR-measured glycoproteins were significantly positively associated with FLI, ALT and GGT (p<0.05). After adjusting for sex, age and BMI, all TRL and glycoprotein correlations remained significant, while all hsCRP correlations, except for GGT, were lost. Likewise, Glyc-A showed a strong positive association with plasma TGs, non-HDL-C and Apo B-100 (ρ=0.804, ρ=0.479 and ρ=0.390, p<0.001 respectively), whereas no significant association was found with LDL-C. Similar associations were also observed with Glyc-B (ρ=0.583, p<0.001 for TGs; ρ=0.239, p<0.001 for non-HDL-C, and ρ=0.186, p=0.002 for Apo B-100).

**Table 2:** Univariate associations of lipid-associated plasma parameters, TRL-P and acute phase proteins with standard fatty liver markers.

Variable	FLI		FIB-4		AST		ALT		GGT	
	ρ (rho)	p value	ρ (rho)	p value	ρ (rho)	p value	ρ (rho)	p value	ρ (rho)	p value
Triglycerides	0.492	<0.001*	-0.159	0.008	0.317	<0.001*	0.369	<0.001*	0.334	<0.001*
LDL-C	-0.084	0.171	0.097	0.104	-0.106	0.077	-0.179	0.003	0.016	0.789
Non-HDL-C	0.174	0.004*	0.022	0.710	0.131	0.029*	0.071	0.237	0.188	0.002*
Apo B-100	0.125	0.041*	0.034	0.570	0.057	0.344	0.001	0.993	0.166	0.006*
Large TRL-P	0.441	<0.001*	-0.126	0.035	0.300	<0.001*	0.343	<0.001*	0.287	<0.001*
Medium TRL-P	0.425	<0.001*	-0.083	0.169	0.316	<0.001*	0.326	<0.001*	0.288	<0.001*
Small TRL-P	0.422	<0.001*	-0.087	0.147	0.287	<0.001*	0.315	<0.001*	0.283	<0.001*
Total TRL-P	0.425	<0.001*	-0.088	0.144	0.293	<0.001*	0.318	<0.001*	0.284	<0.001*
Glyc-A	0.423	<0.001*	-0.070	0.244	0.239	<0.001*	0.246	<0.001*	0.277	<0.001*
Glyc-B	0.427	<0.001*	-0.140	0.019	0.115	0.056	0.184	0.002*	0.285	<0.001*
hsCRP	0.317	<0.001	-0.033	0.644	0.097	0.178	0.130	0.069	0.330	<0.001*

Spearman correlation coefficients (rho) and significance (P-values). *Remained significance after adjustment for sex, age and BMI.

### TRL and Glycoprotein Profile in Ultrasound-Confirmed Hepatic Steatosis

**Figure 2:**
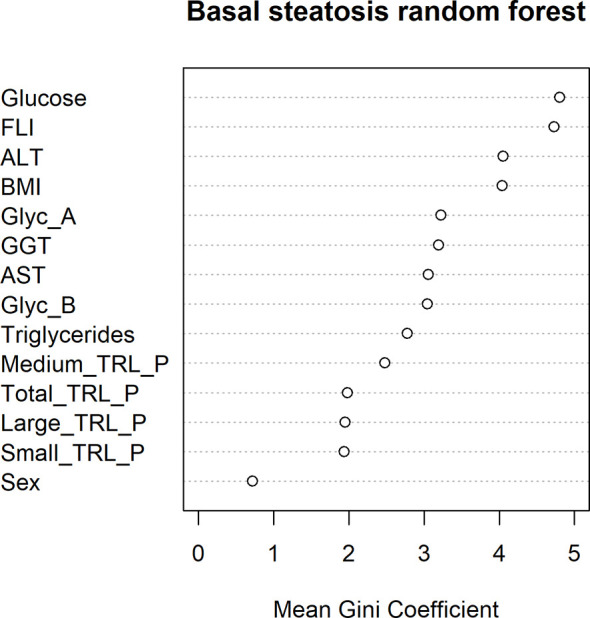
Random forest mean Gini Coefficient for each variable in order to study their importance in the classification of ultrasound-confirmed hepatic steatosis.

The presence of hepatic steatosis was assessed by ultrasound echography in 100 patients in our cohort. Hepatic steatosis was present in 34% of the subjects studied. A higher prevalence of Type 2 Diabetes was observed among the patients who presented hepatic steatosis (p=0,034). Steatosis-free patients showed significantly lower levels of fasting plasma glucose (p=0.04), plasma TG and TRL particles (p<0.01), as well as lower levels of AST, ALT and FLI (p<0.05). We observed a nonsignificant trend towards higher NMR-glycoprotein concentrations in the group with hepatic steatosis, as confirmed by ultrasound. After adjusting by sex, age and BMI, the variables that remained significantly associated with hepatic steatosis were triglycerides, TRL-P subclasses, AST, ALT, GGT and FLI (**Table 3**). In addition, the mean decrease Gini plot from the random forest analysis showed that TRL particles, Glyc-A and B, and transaminases were determinant in order to classify patients with or without ultrasound-confirmed hepatic steatosis (**Figure 2**).

**Table 3:** Clinical and biochemical parameters of 100 ultrasound-studied patients sorted by the presence (YES) or absence (NO) of hepatic steatosis.

	ULTRASOUND-CONFIRMED HEPATIC STEATOSIS			
	NO (n=66)	YES (n=34)	P value Univariate	P value* Multivariate
Age, years	63 (53-69)	62 (56-66)	0.716	–
Sex, male (%)	38.8	67.6	**0.006**	**-**
BMI, kg/m^2^	29.78 (27,8-33,33)	31.14 (28,87-35,5)	**0,035**	**-**
Type 2 Diabetes, %	65.7	85.3	**0.037**	**-**
Insulin therapy, %	20.6	11.8	0.332	–
Oral antidiabetic therapy, %	43.3	61.8	0.079	–
Statins therapy, %	49.2	61.8	0.234	–
Hypotensors therapy, %	53.7	58.8	0.627	–
Triglycerides, mmol/L	1.86 (1.28-3.16)	2.85 (2.03-4.59)	**0.009**	**0.012**
LDL-C, mmol/L	3.85 ± 1.31	3.63 ± 1.16	0.397	0.397
Non-HDL-C, mmol/L	4.74 ± 1.34	4.83 ± 1.20	0.740	0.660
Apo B-100, mg/dL	129.23 ± 34.19	132.15 ± 30.12	0.675	0.677
Glucose, mg/dL	121 (101-157)	138 (119-155)	**0.041**	0.094
HbA_1c_, %	5.90 (5.30-6.90)	6.40 (5.70-7.05)	0.249	0.457
Total TRL-P (nmol/L)	65.81 (45.74-107.8)	96.7 (71.03-149.71)	**0.007**	**0.006**
Large TRL-P (nmol/L)	1.48 (1.02-2.34)	2.33 (1,62-3.27)	**0.004**	**0.003**
Medium TRL-P (nmol/L)	8.29 (5.66-14.5)	13.26 (9.5-22.29)	**0.006**	**0.004**
Small TRL-P (nmol/L)	56.96 (39.02-88.19)	81.14 (61.52-124.26)	**0.008**	**0.008**
AST, U/L	22 (20-26)	25 (22-37)	**0.041**	**0.019**
ALT, U/L	17 (12-25)	23.5 (15-40)	**0.010**	**0.002**
GGT, U/L	22 (15-38)	27 (20-46)	0.069	**0.002**
FLI, %	72.05 (41.27-92.98)	86.18 (77.49-96.02)	**0.002**	**<0.001**
FIB-4	1.73 ± 0.48	1.7 ± 0.39	0.748	0.685
Glyc-A, µmol/L	885.1 (769.5-1126.4)	1021.4 (913.3-1212.1)	0.078	0.082
Glyc-B, µmol/L	361.6 (327.6-412.2)	370.5 (338.9-427.8)	0.395	0.547
hsCRP	2.17 (1.19-3.33)	2.31 (1.42-3.09)	0.801	0.809

Data are the means ± SD for normally distributed variables, medians (IQR) for nonparametric data or n (%). BMI, body mass index; LDL-C, LDL cholesterol; non-HDL-C, non-HDL cholesterol; Apo B-100, apolipoprotein B100; HbA_1c_, glycated hemoglobin; AST, aspartate aminotransferase; ALT, alanine aminotransferase; GGT, gamma-glutamyl transferase; FLI, Fatty Liver Index; FIB-4, Fibrosis-4 score; TRL, triglyceride rich lipoprotein; hsCRP, high-sensitivity C-reactive protein. p values are for group comparisons. Statistical univariate analysis: χ2 for categorical data; t-tests or Mann-Whitney U tests were used for the continuous variables. Statistical linear multivariate analysis controlled by age, sex and BMI. p values* are for group comparisons. Bold values indicate p < 0.05.

No differences were observed in the associations found between NMR-glycoproteins and TRL-P when the hepatic steatosis studied patients were stratified by glucose levels (glucose ≤ 126 mg/dL and glucose > 126 mg/dL) (**Supplementary Table 2**). We did not find significant associations between glycoprotein levels and the presence or not of hepatic steatosis when patients were sorted by glucose levels (**Supplementary Table 3**).

### Glycoprotein Profile and TRL-P in the Prospective Study for the Development of Ultrasound-Confirmed Hepatic Steatosis

After a 10-year follow-up, 18 (28.13%) new cases of hepatic steatosis were confirmed among 64 patients who were disease-free at baseline. **Table 4** summarizes the baseline levels of clinical and standard hepatic biomarkers in both groups (disease-free and ultrasound-confirmed steatosis after 10 years). Both groups were age and sex balanced. The mean age of the study subjects was 63 (53-69) years, and 61.2% were women. As shown in **Table 4**, baseline plasma TG, AST, ALT, GGT and FLI were significantly higher in the group that developed ultrasound-confirmed steatosis (p<0.05). No differences between groups were seen for FIB-4 or hsCRP.

**Table 4:** Baseline clinical and biochemical parameters of ultrasound-studied patients after a 10-year follow-up, grouped as disease-free (NO) or ultrasound-confirmed hepatic steatosis (YES).

	ULTRASOUND-CONFIRMED HEPATIC STEATOSIS			
	NO (n=46)	YES (n=18)	P value Univariate	P value* Multivariate
Age, years	64 (56-69)	57 (50-69)	0.209	–
Sex, male (%)	34.8	52.6	0.182	–
BMI, kg/m^2^	29.72 (27.7-33.42)	30.29 (28.69-33)	0.599	–
Type 2 Diabetes, %	65.2	68.4	0.804	–
Incident Type 2 Diabetes, %	0	25	0.576	–
Incident CVD, %	17.4	5.9	0.247	–
Insulin therapy, %	21.1	10.5	0.289	–
Oral antidiabetic therapy, %	47.8	36.8	0.418	–
Statins therapy, %	52.2	42.1	0.460	–
Hypotensors therapy, %	54.3	52.6	0.900	–
Triglycerides, mmol/L	1.73 (1.21-2.93)	2.49 (1.79-4.27)	**0.029**	**0.007**
LDL-C, mmol/L	3.83 ± 1.19	3.95 ± 1.65	0.742	0.526
Non-HDL-C, mmol/L	4.58 ± 1.22	5.21 ± 1.57	0.086	0.360
Apo B-100	126.36 ± 30.43	140.94 ± 40.51	0.124	0.563
Glucose, mg/dL	117 (96-157)	132 (104-179)	0.175	**0.045**
HbA_1c_, %	6 (5.45-6.90)	5.50 (5.20-7.10)	0.472	0.782
ALT, U/L	18.5 (14-28)	25 (19-43)	**0.004**	**<0.001**
GGT, U/L	18.5 (14-28)	46 (23-173)	**<0.001**	**<0.001**
FLI, %	68.1 (40.5-86.7)	82.97 (63.1-96.2)	**0.019**	**<0.001**
FIB-4	1.71 ± 0.41	1.78 ± 1.13	0.595	0.587
hsCRP	2.18 ± 1.09	2.42 ± 1	0.416	0.200

Data are the means ± SD for normally distributed variables, medians (IQR) for nonparametric data or n (%). BMI, body mass index; Incident CVD, incident cardiovascular disease; LDL-C, LDL cholesterol; non-HDL-C, non-HDL cholesterol; Apo B-100, apolipoprotein B100; HbA_1c_, glycated hemoglobin; AST, aspartate aminotransferase; ALT, alanine aminotransferase; GGT, gamma-glutamyl transferase; FLI, Fatty Liver Index; FIB-4, Fibrosis-4 score; hsCRP, high-sensitivity C-reactive protein. p values are for group comparisons. Statistical univariate analysis: χ2 for categorical data; t-tests or Mann-Whitney U tests were used for continuous variables. Statistical linear multivariate analysis controlled by age, sex and BMI. p values* are for group comparisons. Bold values indicate p < 0.05.

**Figure 3:**
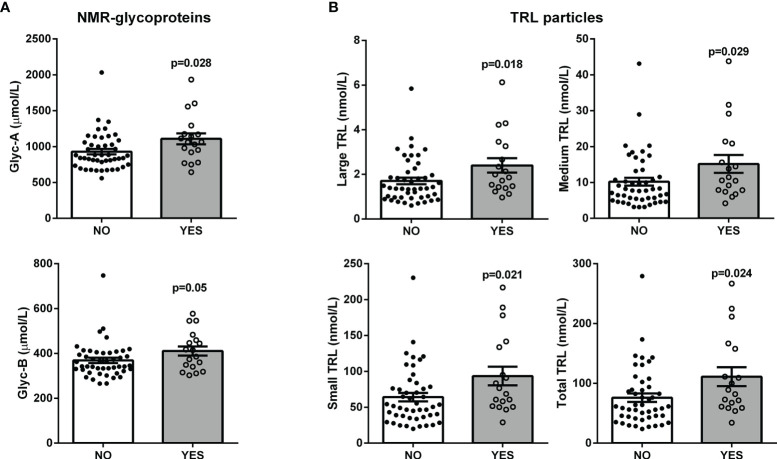
Scatter plot with bar graphs of the plasma baseline levels of the NMR-measured glycoproteins **(A)** and TRL particles **(B)** in patients with ultrasound-confirmed hepatic steatosis after a 10-year follow-up. Each dot represents a patient: black dots for disease-free patients and white dots for patients with steatosis; bars represent mean values; p-values from Mann-Whitney U tests. Linear multivariate analysis controlled by age, sex and BMI: Glyc-A, p = 0.062; Glyc-B = 0.289; Large TRL-P, p = 0.001; Medium TRL-P, p = 0.004; Small TRL-P, p = 0.003; Total TRL-P, p = 0.003.

**Figure 4:**
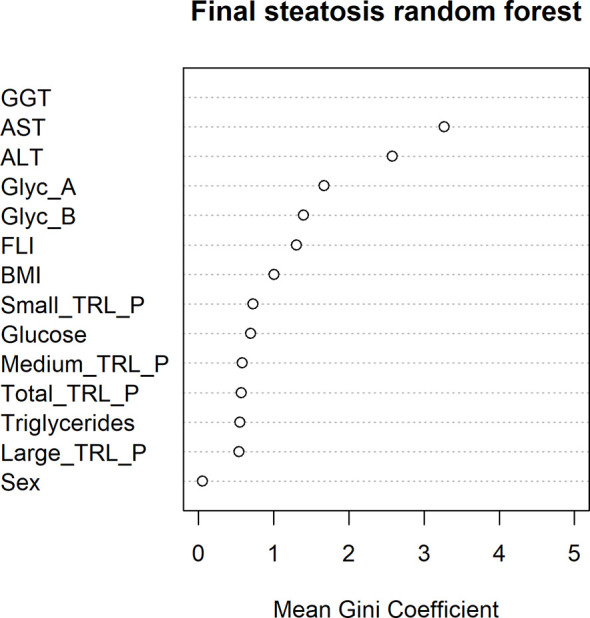
Random forest mean Gini Coefficient for each variable in order to study their importance in the classification of ultrasound-confirmed hepatic steatosis after a 10 years’ follow-up.

Interestingly, baseline levels of the NMR-measured TRL particles and glycoproteins were higher in the group of 18 patients who developed hepatic steatosis after 10 years (p ≤ 0.05) (**Figure 3**). After adjusting by sex, age and BMI, triglycerides, TRL-P subclasses, AST, ALT, GGT and FLI remained significantly associated with hepatic steatosis. The mean decrease Gini plot from the random forest analysis showed that TRL particles, Glyc-A and B and transaminases were determinant in the classification of patients with hepatic steatosis (**Figure 4**).

## Discussion

In the present study, we cross-sectionally characterized the TRL subclass particle number, sorted by size, and the plasma glycoprotein profile measured by ^1^H-NMR in 280 patients at metabolic risk. We detected remarkably significant positive associations between all TRL particle subclasses and Glyc-A and B concentrations. These findings remained robust after correction by different covariates. Likewise, we found positive associations of TRL and Glyc-A and B with hepatic injury-related markers. These correlations were weaker with the liver fibrosis index FIB-4. Liver ultrasound was performed at baseline in 100 patients to evaluate the presence of hepatic steatosis, and we detected higher levels of TRL particles in patients with steatosis. Moreover, all baseline TRL subclasses and Glyc-A and B were higher, although well within the normal range, in those subjects developing fatty liver during follow-up. Interestingly, hsCRP concentrations were not associated with TRL particles or liver alteration biomarkers.

^1^H-NMR allows the detection of changes in the number and size of lipoprotein subclasses early either in nutritional and pharmacological intervention studies (29, 30). In this study, we measured the particle number of three VLDL subclasses by ^1^H-NMR to assess relative differences among larger vs smaller VLDL. Moreover, we studied the association of these different VLDL particle subclasses with inflammatory markers synthesized in the liver (Glyc-A and B), hsCRP and standard clinical indexes and imaging data of liver steatosis.

In our study, we found a strong and positive association between all TRL particle subclasses and ^1^H-NMR-measured glycoproteins Glyc-A and Glyc-B. Age, BMI and sex are known factors that influence triglyceride concentrations (31). In our study, the associations found between TRL and glycoproteins remained positive when controlling for these covariates but also remained robust when adjusting by different covariates found to be related in the multivariate regression analysis. Recent studies have shown the implications of these glycoprotein markers in inflammatory and autoimmune diseases and in conditions such as rheumatoid arthritis, polycystic ovary syndrome and HIV infection, among others (18–20). Glyc-A has been consistently associated with systemic inflammation and atherosclerotic cardiovascular disease (17, 32–34), as well as with T2DM (35). Nevertheless, our group has previously reported the implications of the other glycoprotein marker Glyc-B in HIV and systemic inflammation (16, 19). Interestingly, in the present study, we found that all of the parameters measured (Glyc-A and Glyc-B) showed strong associations with TRL-P. This observation highlights the association of triglycerides with inflammation. Although T2DM-associated hypertriglyceridemia is due to the hypersecretion of large TRLs, in our hands, all particle subgroups, characterized by NMR, were equally associated with inflammation parameters. Interestingly, we detected higher baseline concentrations of these markers in 18 patients who developed steatosis over a 10-year follow-up study, suggesting that subclinical alterations could be present many years before its clinical manifestation.

Additionally, in our cohort of patients at metabolic risk, we found that TRL particles and NMR-glycoproteins had a positive association with hepatic dysfunction markers, including the fatty liver index (FLI) and ALT, AST and GGT, serum markers that have been used extensively to generate multiple scores and indexes for the non-invasive assessment of fatty liver disease (26, 27, 36–40). However, their potential for distinguishing or staging more severe conditions, such as steatohepatitis and/or chronic liver disease, is limited. Interestingly, the association between TRL, Glyc-A and B and the clinical indexes of fatty liver disease was weaker or even null for FIB-4. FIB-4 is considered to be a fibrosis marker rather than an inflammation marker, which could explain this result (41).

We showed that patients diagnosed by ultrasound echography with hepatic steatosis had higher serum concentrations of TRL particles. These data agree with previously published data, were triglycerides levels and VLDL particle number were associated with hepatic steatosis (4). Furthermore, although NMR-glycoproteins did not show a significant association with hepatic steatosis, random forest analysis shows them as important variables to discriminate patients with hepatic steatosis.

As already stated, glycoproteins are known to be associated with diabetes (42). We analysed the associations between glycoproteins and TRL and hepatic steatosis stratifying by glucose levels, finding no differences regardless of glucose levels.

Plasma glycoproteins measured by ^1^H-NMR belong to the family of acute phase proteins (14, 43) released under systemic inflammatory conditions. Under these states, plasma proteins show an increase in their glycosylated forms by increasing their oligosaccharide ramifications and monosaccharide residues (42, 44). In our study, we showed that the measurement of these glycoproteins by NMR provides a wider view of the systemic inflammatory status than the measurement of a single marker, hsCRP. Hence, the detection of these glycoforms, which are mainly produced by the liver, could reflect the consequence of (1) a liver overloaded with fat (ectopic fat accumulation), usually associated with lipotoxic fatty molecules that cannot be counterbalanced by (2) the overproduction of VLDL particles, as happens in hepatic steatosis (3, 4, 45). In accordance, given the implications of inflammation in the progression of MAFLD and the chronic inflammatory status present in metabolic syndrome and related conditions (including obesity, insulin resistance and T2DM), the role of these glycoproteins could be of potential interest in the detection of such an inflammatory status in fatty liver disease (16, 19). In addition, MAFLD has been reported to be associated with increased CVD morbidity and mortality, making its detection even more clinically important (46).

For the prospective part of this study, we evaluated new-onset liver steatosis in 64 patients without fatty liver at baseline by ultrasound. We compared baseline levels of TRL-P and glycoproteins between those who had developed steatosis and those who remained disease-free after 10 years. First, all baseline TRL-P concentrations were higher in those who developed steatosis, and their baseline glycoprotein levels were higher. Multivariate models also confirmed the associations between TRL particles and hepatic steatosis and established the glycoprotein parameters as determinant classifiers.

Some limitations of our study must be pointed out. First, our findings are based on associations and correlations, limiting the explanation of the possible causal molecular mechanisms. For the detection of hepatic steatosis, we used standard biochemical markers and clinical indexes; in addition, ultrasonography has the limitation that it can only detect steatosis with >2.5%–20% liver fat content and, therefore, a relevant number of patients with steatosis starting at 5% of liver fat content can be missed (47–49). Unfortunately, we did not have access to liver biopsies of our patients, the gold-standard technique for fatty liver disease diagnosis. Furthermore, the sample size of our follow-up study is limited; however, the follow-up period of 10 years strengthens our findings. The main strength of our study is that we used ^1^H-NMR lipoprotein and glycoprotein profiling, providing a wide view of different plasma parameters, which can be interpreted as a molecular signature of the metabolic and inflammatory status of our patients. Indeed, the “Glyc” NMR-measured signals determine the plasma levels of various acute-phase glycoproteins, giving information about the overall inflammatory state rather than relying on the measurement of a single reactant, such as C-reactive protein (hsCRP).

In conclusion, the characterization of TRL subgroup particles and glycoprotein A and B concentrations by ^1^H-NMR of patients at metabolic risk provides information on the inflammatory status that accompanies metabolic syndrome as well as its relationship with alterations in the liver. We found no differences in the distribution of VLDL particle subclasses, according to size, in this group of patients. In addition, we show evidence supporting that the measurement of baseline TRL particles and plasma glycoproteins could have predictive value for the development of MAFLD and its complications. This study could provide new insights into the use of NMR spectroscopy for the development of new lipidic and glycoprotein-related biomarkers for hepatic disease.

## Data Availability Statement

The raw data supporting the conclusions of this article will be made available by the authors, without undue reservation.

## Ethics Statement

The studies involving human participants were reviewed and approved by the Ethical and Clinical Investigation Committee of the Pere Virgili Institute for Health Research (IISPV) and fulfilled the principles of the Helsinki Declaration. The patients/participants provided their written informed consent to participate in this study.

## Author Contributions

JM-V: study design, data analysis and interpretation, drafting of the manuscript, review of the results, review of the manuscript. RR, EO, DL, ML, MB, RR-C, NP, AP and AC: data analysis, review of the results. DI: study design, review of the results, and review of the manuscript. JG: study design, data analysis and interpretation, drafting of the manuscript, review of the results, review of the manuscript. LM: study design, data analysis and interpretation, review of the results, review of the manuscript, overall study oversight and guarantor of the manuscript. All authors contributed to the article and approved the submitted version.

## Funding

This study was funded by grants from Instituto de Salud Carlos III (ISCIII), Madrid, Spain (PI18/00515). This work was jointly supported by national funding from the Spanish Biomedical Research Centre in Diabetes and Associated Metabolic Disorders (CIBERDEM). This work was co-funded by the European Regional Development Fund (ERDF).

## Conflict of Interest

EO works at Biosfer Teslab.

The remaining authors declare that the research was conducted in the absence of any commercial or financial relationships that could be construed as a potential conflict of interest.

## Publisher’s Note

All claims expressed in this article are solely those of the authors and do not necessarily represent those of their affiliated organizations, or those of the publisher, the editors and the reviewers. Any product that may be evaluated in this article, or claim that may be made by its manufacturer, is not guaranteed or endorsed by the publisher.
