# Supplementary material for: Triglyceride-Rich Lipoproteins and Glycoprotein A and B Assessed by 1H-NMR in Metabolic-Associated Fatty Liver Disease
Source: Front Endocrinol (Lausanne). 2022 Jan 10;12:775677. doi: 10.3389/fendo.2021.775677 (PMC8785395; doi:10.3389/fendo.2021.775677)
Supplement: Supplementary file 1 [file Table_1.docx]

**Supplementary Table 1.** Multivariate linear regression analysis for TRL-P and glycoproteins.

| *Dependent variables* | Large TRL-P | | | Medium TRL-P | | | Small TRL-P | | | Total TRL-P | | |
| --- | --- | --- | --- | --- | --- | --- | --- | --- | --- | --- | --- | --- |
|  | B | p | R^2^ | B | p | R^2^ | B | p | R^2^ | B | p | R^2^ |
| *Independent variables:* |  |  |  |  |  |  |  |  |  |  |  |  |
| Glyc-A, µmol/L |  |  |  |  |  |  |  |  |  |  |  |  |
| Model 1: crude | **0.004** | **<0.001** | **0.790** | **0.027** | **<0.001** | **0.777** | **0.146** | **<0.001** | **0.842** | **0.177** | **<0.001** | **0.839** |
| Model 2: adjusted | **0.004** | **<0.001** | **0.830** | **0.028** | **<0.001** | **0.803** | **0.147** | **<0.001** | **0.859** | **0.178** | **<0.001** | **0.858** |
| Glyc-B, µmol/L |  |  |  |  |  |  |  |  |  |  |  |  |
| Model 1: crude | **0.011** | **<0.001** | **0.564** | **0.088** | **<0.001** | **0.571** | **0.463** | **<0.001** | **0.590** | **0.563** | **<0.001** | **0.593** |
| Model 2: adjusted | **0.011** | **<0.001** | **0.638** | **0.084** | **<0.001** | **0.637** | **0.438** | **<0.001** | **0.675** | **0.533** | **<0.001** | **0.677** |

Linear regression analysis results are displayed as beta coefficients with the p value and R^2^ values for each model. Large, medium, small and total TRL-P were the dependent variables. Model 1 refers to each glycoprotein alone as independent variable and Model 2 includes age, BMI, hsCRP, ALT, AST, GGT, systolic BP, glucose, total cholesterol, and sex.
