# Supplementary material for: Triglyceride-Rich Lipoproteins and Glycoprotein A and B Assessed by 1H-NMR in Metabolic-Associated Fatty Liver Disease
Source: Front Endocrinol (Lausanne). 2022 Jan 10;12:775677. doi: 10.3389/fendo.2021.775677 (PMC8785395; doi:10.3389/fendo.2021.775677)
Supplement: Supplementary file 2 [file Table_2.docx]

**Supplementary Table 2. Univariate associations of NMR-glycoproteins with TRL-P stratified by glucose tolerance.**

| *Variable* | Large TRL-P | | Medium TRL-P | | Small TRL-P | | Total TRL-P | |
| --- | --- | --- | --- | --- | --- | --- | --- | --- |
|  | ρ (rho) | p value | ρ (rho) | p value | ρ (rho) | p value | ρ (rho) | p value |
| *Glucose ≤ 126 mg/dL (n=104)* | | | | | | | | |
| Glyc-A | 0.864 | <0.001 | 0.897 | <0.001 | 0.886 | <0.001 | 0.890 | <0.001 |
| Glyc-B | 0.657 | <0.001 | 0.657 | <0.001 | 0.654 | <0.001 | 0.657 | <0.001 |
| *Glucose > 126 mg/dL (n=175)* | | | | | | | | |
| Glyc-A | 0.856 | <0.001 | 0.893 | <0.001 | 0.897 | <0.001 | 0.898 | <0.001 |
| Glyc-B | 0.631 | <0.001 | 0.636 | <0.001 | 0.655 | <0.001 | 0.654 | <0.001 |

Spearman correlation coefficients (rho) and significance (*P*-values).
