# Supplementary material for: Triglyceride-Rich Lipoproteins and Glycoprotein A and B Assessed by 1H-NMR in Metabolic-Associated Fatty Liver Disease
Source: Front Endocrinol (Lausanne). 2022 Jan 10;12:775677. doi: 10.3389/fendo.2021.775677 (PMC8785395; doi:10.3389/fendo.2021.775677)
Supplement: Supplementary file 3 [file Table_3.docx]

**Supplementary Table 3. NMR-glycoproteins levels of 100 ultrasound-studied patients sorted by the presence (YES) or absence (NO) of hepatic steatosis and stratified by glucose levels.**

| Ultrasound-confirmed hepatic steatosis | | | |
| --- | --- | --- | --- |
|  | No | Yes | P-value |
|  | *Glucose ≤ 126 mg/dL* | |  |
| *n* | 34 | 10 |  |
| Glyc-A (µmol/L) | 840,04 (714,58-1061,92) | 1087,57 ± 287,03 | 0.075 |
| Glyc-B (µmol/L) | 363,23 ± 59,16 | 384,68 ± 54,85 | 0.312 |
|  | *Glucose > 126 mg/dL* | |  |
| *n* | 32 | 24 |  |
| Glyc-A (µmol/L) | 1035,68 ± 326,83 | 1019,25 (906,32-1126,11) | 0.675 |
| Glyc-B (µmol/L) | 361,61 (332,15-439,35) | 387,47 ± 87,64 | 0.954 |

Data are the means ± SD for normally distributed variables, medians (IQR) for nonparametric data or n (%). The stratification by glucose tolerance was performed according to plasma glucose levels *≤* 126 mg/dL for normo- and pre-diabetic patients and >126 mg/dL for diabatic patients. Statistical analysis: t-tests or Mann-Whitney U tests were used for the continuous variables.
